# Microstructural, Mechanical, and Electrochemical Characterization of CrMoNbTiZr High-Entropy Alloy for Biomedical Application

**DOI:** 10.3390/ma16155320

**Published:** 2023-07-28

**Authors:** Akeem Akinwekomi, Farid Akhtar

**Affiliations:** 1 Division of Materials Science, Luleå University of Technology, 97187 Luleå, Sweden; akeem.akinwekomi@ltu.se; 2 Department of Metallurgical and Materials Engineering, Federal University of Technology Akure, Akure 340252, Ondo State, Nigeria

**Keywords:** high-entropy alloy, biomaterials, CrMoNbTiZr, powder methods, bio-corrosion

## Abstract

High-entropy alloys (HEA) with superior biocompatibility, high pitting resistance, minimal debris accumulation, and reduced release of metallic ions into surrounding tissues are potential replacements for traditional metallic bio-implants. A novel equiatomic HEA based on biocompatible metals, CrMoNbTiZr, was consolidated by spark plasma sintering (SPS). The relative sintered density of the alloy was about 97% of the theoretical density, indicating the suitability of the SPS technique to produce relatively dense material. The microstructure of the sintered HEA consisted of a BCC matrix and Laves phase, corresponding to the prediction of the thermodynamic CALPHAD simulation. The HEA exhibited a global Vickers microhardness of 531.5 ± 99.7 HV, while the individual BCC and Laves phases had hardness values of 364.6 ± 99.4 and 641.8 ± 63.0 HV, respectively. Its ultimate compressive and compressive yield strengths were 1235.7 ± 42.8 MPa and 1110.8 ± 78.6 MPa, respectively. The elasticity modulus of 34.9 ± 2.9 GPa of the HEA alloy was well within the range of cortical bone and significantly lower than the values reported for commonly used biomaterials made from Ti-based and Cr–Co-based alloys. In addition, the alloy exhibited good resistance to bio-corrosion in PBS and Hanks solutions. The CrMoNbTiZr HEA exhibited an average COF of 0.43 ± 0.06, characterized mainly by abrasive and adhesive wear mechanisms. The CrMoNbTiZr alloy’s mechanical, bio-corrosion, and wear resistance properties developed in this study showed a good propensity for application as a biomaterial.

## 1. Introduction

Aging populations, increasing road/industrial accidents, and rising cases of chronic diseases have contributed to the increased demand for biomedical implants [1]. Among all the different types of implant products, the largest market share, of about 38%, is controlled by orthopedics and trauma-based medical implants [1]. Biomedical implants are used for different applications, including body part replacement, augmentation of body functions, and supporting body tissues and organs. Implants are expected to exhibit high corrosion and wear resistance and should elicit appropriate responses from the surrounding tissues [2]. Commonly used metallic biomaterials are derived from magnesium (Mg)-based, titanium (Ti)-based, 316L stainless steel, nickel–titanium (NiTi), and cobalt–chromium–molybdenum (CoCrMo) alloys. Mg is particularly attractive because of its biocompatibility, low density, and elastic modulus of 41–45 GPa, close to those of human cortical bones [3]. Moreover, Mg^2+^ formed from the degradation of Mg-based implants is beneficial for bone regeneration [4]. Nonetheless, some technical challenges militating against the application of Mg-based materials include their rapid degradation rate in a physiological environment [5] and the accumulation of large amounts of hydrogen gas, which can lead to wound interface cavitation and tissue necrosis [5]. Traditionally, 316L has been used for relatively cheap implant applications, with suitable biocompatibility and good mechanical properties. Nevertheless, it suffers from pitting corrosion, low wear resistance, debris accumulation [6], and release of Ni and Co ions, which have been implicated in metal allergy [7] and dermatitis [8]. Ti6Al4V is a Ti-based alloy used as a biomedical implant due to its high biocompatibility and good mechanical and high electrochemical characteristics. However, it exhibits a low wear resistance and has an elastic modulus higher than bones, which may cause stress shielding or loosening of implants [9]. Long-term use of Ti6Al4V alloys is implicated in releasing Al and V ions, which cause different health problems, such as metal allergy [2,10]. Further, the ions can weaken interfacial bonding between the implant and the surrounding bones, negatively impacting osseointegration [11]. A recent study advocates the use of Ti–Mo-based biomaterials due to their high cytocompatibility and enhanced osseointegration [12]. Similarly, CoCrMo alloys exhibit an excellent combination of strength and corrosion resistance [9]. Still, they are known to suffer from the accumulation of wear debris, especially in bearing applications, such as hip and knee replacement prostheses. This debris increases the concentrations of Co and Cr in the blood and serum of patients [10]. Their ions are believed to be carcinogenic and to promote inflammation [8,10].

Therefore, it is imperative to develop new metallic biomaterials. High-entropy alloys (HEAs) have been investigated in this regard. These are metallic alloys that contain five or more multi-principal elements whose composition varies between 5 and 35 atomic weight percent [13,14,15]. The choice of metals and the mass addition of each metal in the HEA gives an almost limitless compositional range of potential metallic alloys with the potential to tailor mechanical, corrosion, and wear properties for biomedical alloys. HEAs exhibit high configurational entropy that favors the formation of a single-phase solid solution. Moreover, the lattice distortion and sluggish diffusion characteristics are responsible for excellent mechanical properties [13,14,15]. Various groups of metallic elements, such as transitional (Fe, Co, Cr, Ni, Mn) [16,17,18] and refractory (Ti, Zr, Ta, Nb, Mo, V, etc.) [19,20,21,22,23], have been used to develop HEAs. Specific to biomedical applications, Ti–Zr-based HEAs have been investigated due to their biocompatibility, hardness, strength, and good wear resistance. Motallebzadeh et al. investigated the mechanical and corrosion behaviors of TiZrTaHfNbZr and non-equimolar Ti1.5ZrTa0.5Hf0.5Nb HEAs and showed that the HEAs exhibited better corrosion resistance than 316L, CoCrMo and Ti6Al4V [21]. Similarly, Ti40Zr20Hf20Fe20 had enhanced corrosion resistance and cell adhesion, proliferation, and differentiation properties compared to Ti6Al4V. The HEA sample showed lower Young’s and shear moduli than the reference Ti6Al4V [2]. Several other HEA systems demonstrated superior corrosion resistance and lower wear rates than existing Ti6Al4V, 316L SS, and CoCrMo alloys in various physiological fluids [24,25,26].

Most biomedical HEAs have been processed via ingot metallurgy through the arc melting technique. However, due to the high melting points of the refractory elements utilized in these HEAs, incomplete melting and chemical heterogeneity [22], as well as casting defects and a coarse microstructure [27,28] may develop in the alloys. In addition, the processing route may not be compatible with industrial processes due to the high cost of equipment and limitations in product shape and size [29]. Powder metallurgy (PM) is advantageous due to its near-net shape capability and competence in the processing of refractory and other elements with different densities [7,30,31,32] to minimize evaporation of low-melting point elements. The PM process can also produce non-equilibrium phases, and a more homogenous and nanocrystalline grain structure [33,34]. Some recent studies demonstrated that the mechanical properties of PM-processed refractory HEAs were at least at par or superior to those obtained by ingot metallurgy [27,28,35]. However, only a handful of studies have investigated the electrochemical properties of these biomedical PM-processed HEAs. Xiang et al. investigated the effect of sintering temperatures on the compression, hardness, and electrochemical behaviors of PM-processed NbTaTiZr refractory HEA [35]. They showed that samples sintered at 800 °C possessed the highest microhardness, of 662 HV, while the highest compressive yield strength (2378 MPa) and modulus (141 GPa) were exhibited by samples sintered at 900 °C. Although samples sintered between 700 and 900 °C were susceptible to pitting corrosion in Hanks’ solution, higher sintering temperatures of 1000 and 1100 °C yielded pitting-resistant alloys comparable to those of commercially pure Ti and Ti6Al4V [35].

Herein, CALPHAD thermodynamics-based simulation was used to design, predict the phases, and select the sintering temperature of an equiatomic CrMoNbTiZr HEA. Several important factors were considered in selecting the constituent elements. All the elements have good biocompatibility and high polarization resistance [7,36], which can lower cytotoxicity [7,36] and enhance corrosion resistance, respectively. These factors are important to reduce the incidence of revision surgery required to replace damaged implants [36]. Moreover, the refractory properties of the elements, such as high strength, shear modulus, and hardness may minimize wear, debris formation, and metal allergies [9,37]. Mo has been reported to significantly enhance solid solution formation [37], while Nb induces the transformation of BCC to the Laves phase to enhance the microhardness and wear resistance of some HEAs [38,39]. Therefore, in this study, the CrMoNbTiZr HEA alloy was synthesized by elemental powder mixing and consolidated via the spark plasma sintering technique. The alloy’s microstructure, hardness, compression, wear, and electrochemical behaviors were investigated. The microstructure comprised mainly BCC and Laves phases, which resulted in high microhardness and compressive properties. Significantly, the elastic modulus of the alloy was within the range reported for cortical bone and significantly lower than the values reported for some commonly used biomaterials made from Ti-based and Cr–Co-based alloys. This indicates the potential of the developed alloy for orthopedic applications. Additionally, the alloy exhibited good resistance to bio-corrosion in simulated physiological solutions. The results were discussed and compared with some of the existing metallic biomaterials.

## 2. Materials and Methods

### 2.1. Design and Synthesis of Alloy

**Figure 1:**
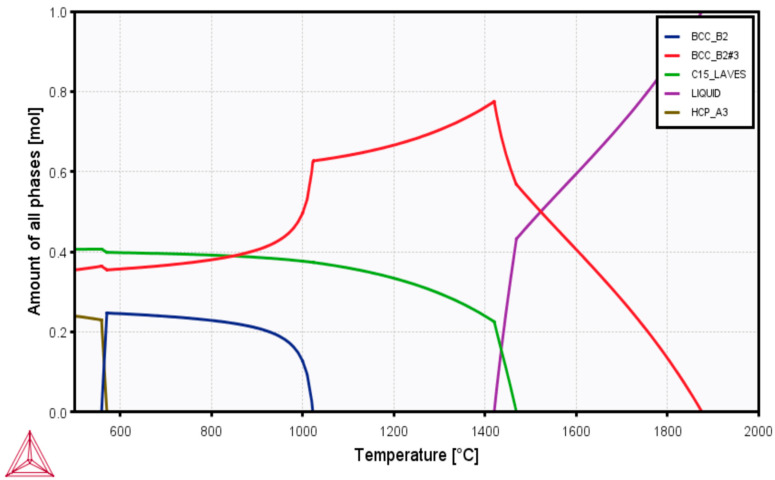
CALPHAD simulation of the phases predicted in equiatomic CrMoNbTiZr HEA.

The principal elements of the HEA design comprised biocompatible elements, including chromium (Cr), molybdenum (Mo), niobium (Nb), titanium (Ti), and zirconium (Zr) in equiatomic concentrations. All the elements have the BCC crystal structure except for Ti and Zr, which have the HCP crystal structure. Furthermore, CALPHAD-based simulation (Figure 1) using the ThermoCalc software (TCHEA3 database) was used to predict the likely phases in the HEA and select a suitable sintering temperature. At temperatures between 1100 and 1400 °C, only the BCC and C15 Laves phases co-exist. However, a liquid phase appears above 1400 °C. Although liquid phase sintering may enhance densification, it increases the propensity for carbide formation due to the chemical reaction between the graphite die and some elements, such as Ti [40,41]. Therefore, the sintering temperature of 1200 °C was selected to minimize carbide formation. At 1200 °C, the approximate predicted amounts of BCC and the Laves phases are 0.65 and 0.35 molar percentages, respectively.

### 2.2. Alloy Preparation

All samples were prepared in an argon-filled glove box to minimize powder oxidation. Cr, Mo, Nb, Ti, and Zr powders were procured from US Research Nanomaterials Inc. with 99.5% purity and −325 mesh. The powders were dry-mixed to obtain an equimolar concentration of CrMoNbTiZr HEA. Mixing was done on a roller mill using silicon nitride balls with a ball-to-powder ratio of 10:1 for 1 h. To minimize oxidation of the powders, mixing was carried out under argon gas protection. The mixed powders were transferred into a graphite die of Ø 12 mm and sintered in a spark plasma sintering facility (SPS, Dr. Sinter 2050, Sumitomo Coal Mining Co., Ltd., Tokyo, Japan) under a uniaxial pressure of 40 MPa. The heating rate was 100 °C/min with a holding time of 15 min at 1200 °C. Sintering was undertaken in a vacuum of <15 Pa.

### 2.3. Alloy Characterization

Sintered HEAs were polished on silicon carbide papers of successively increasing grit sizes. The densities of the sintered samples were determined based on the Archimedes principle and benchmarked against the theoretical density estimated from the rule-of-mixture. A JSM-IT300LV scanning electron microscope (SEM, JEOL GmbH, Freising, Germany) equipped with an energy dispersive X-ray spectroscopy (EDX) was used to characterize the microstructure of the samples. The SEM operating parameters were 20 kV of accelerating voltage, a working distance of 12 mm, and a high probe current of 30 in the high vacuum mode. Phases in the sintered HEA were determined from X-ray diffraction measurements using a PANalytical Empyrean machine operated at 45 kV and 40 mA, utilizing Cu-Kα radiation over a range from 2θ = 10° to 80°, with a scan step size of 0.026. Vickers microhardness (Duramin-40 AC3, Struers, Willich, Germany) of the samples (Ø 12 mm × 3 mm) were obtained at room temperature under an applied load of 500 gf for 15 s. The average and standard deviations of at least ten readings were reported.

Room temperature compression tests were undertaken on a Gleeble 3800 (Dynamic Systems Inc., Dallas, TX, USA) at a strain rate of 0.001 s^−1^. Compression test samples were EDM wire-cut into dimensions Ø 5 mm × 10 mm. Testing was done in triplicate, and the average was reported. Nickel pastes and graphite sheets were placed at the end of each sample to minimize friction and barreling effects. Fractured surfaces were examined under the SEM. Wear characterization of the HEA sample was performed on a Rtec Universal Tribometer (Rtec Universal Tribometer, San Jose, CA, USA). A Ø 9.5 mm alumina ball served as the counter body that was used to exert a normal force of 5 N on the HEA. The sliding speed was 0.1 m/s for a total distance of 200 m. Triplicate wear tests were carried out to obtain an average result. The surface morphology and compositions of the wear scars were analyzed on a JEOL SEM/EDX and an optical surface profiler (Zygo NewView 7300 3D, Zygo Corporation, Middlefield, CT, USA).

The electrochemical tests at ambient temperature were conducted using a standard three-electrode setup in phosphate buffered saline (PBS: 8 g of NaCl, 1.44 g of Na_2_HPO_4_, 0.2 g of KCl, 0.24 g of KH_2_PO_4_ in 1000 mL of distilled water, pH 7.4; Sigma-Aldrich, Darmstadt, Germany) and Hanks’ balanced salt solution (HBSS: 8.0 g of NaCl, 0.4 g of KCl, 0.14 g of CaCl_2_, 0.1 g of MgSO_4_∙7H_2_O, 0.1 g of MgCl_2_∙6H_2_O, 0.06 g of Na_2_HPO_4_∙2H_2_O, 0.06 g of KH_2_PO_4_, 1.0 g of glucose, 0.35 g of NaHCO_3_ in 1000 mL of distilled water; Sigma Aldrich, Darmstadt, Germany) on a Gamry Instruments Reference 3000 (Gamry Instruments, Warminster, PA, USA) instrument. CrMoNbTiZr HEA sintered sample was used as the working electrode, while a platinum sheet and Ag/AgCl saturated with KCl solution were used as the counter and reference electrodes, respectively. Open circuit potential (OCP) was established for 1 h followed by potentiodynamic polarization from −0.3 to + 1.0 V vs. OCP at a scan rate of 0.167 mV/s. The corrosion rate was estimated from the Tafel extrapolation of the potentiodynamic curve. The morphology and composition of the corroded surface of the CrMoNbTiZr HEA sample were assessed on an SEM/EDX.

## 3. Results

### 3.1. Density and Microstructural Characterizations

**Figure 2:**
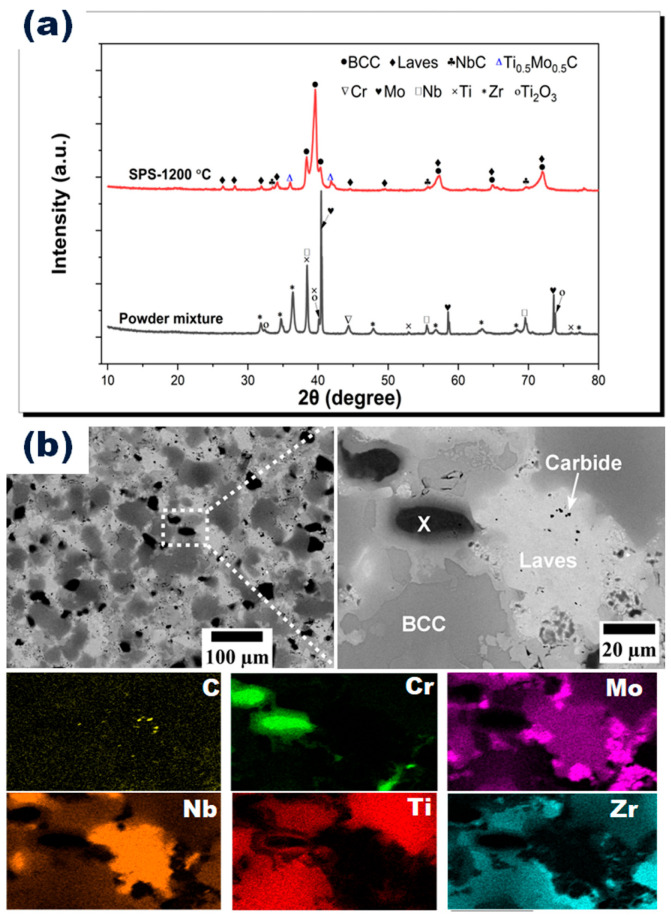
(**a**) XRD diffractograms of the elemental powder mixture and sintered HEA. (**b**) Backscattered electron images and EDX mapping of CrMoNbTiZr HEA.

The sintered density of CrMoNbTiZr HEA was 7.20 g/cm^3^, representing a densification of about 97% compared with the estimated theoretical density of 7.41 g/cm^3^. The selected sintering temperature was adequate to produce relatively dense specimens. Figure 2a shows the X-ray diffractogram of the mixed powder and sintered CrMoNbTiZr HEA. After sintering, peaks of the individual metallic powder components disappeared, leaving only the BCC, Laves, and carbide phases. The Laves phase was identified as Cr_2_Nb (reference code: 01-072-9023), while the carbide phases comprised NbC (reference code: 04-004-7190) and mixed carbides of Ti and Mo (reference code: 04-001-6529). The carbides were formed from the reaction between the metallic elements and the carbon in the graphite die during the sintering process. Similar carbide formation was reported in previous works on SPS-processed HEAs [40,41].

The backscattered electron (BSE) micrograph in Figure 2b shows three contrasts: black, light gray, and dark gray. The black contrast is in two sizes: large black globules (marked X) and tiny black speckles. The large globules (X) were identified in the EDX mapping in Figure 2 as regions enriched in Cr with minor fractions of Nb, Mo, Ti, and C. On the other hand, the light-gray phase consisted primarily of Nb with a minor atomic fraction of Cr and Ti; see Table 1. This phase, corresponding to the Laves phase, was identified from the XRD analysis. In addition, the dark-gray phase was identified as the BCC phase, which contained solid solutions of Ti and Zr with some atomic fractions of Cr, Mo, and Nb. The fine black speckles were identified as carbides from the EDX mapping and were closely associated with Nb, suggesting the formation of NbC. Although the influence of elemental carbides on HEA biocompatibility has not been investigated in the present study, some earlier works have indicated their positive influence on biocompatibility. Enhanced surface stability and osseointegration of TiC coatings on a 316L substrate have been reported [42]. Similarly, NbC coatings have been shown to exhibit superior osteopontin expression (required for cell adhesion and implant integration) compared with a bare Ti6Al4V substrate [43]. Another study suggests that niobium carbide-based hydrogel enhanced antimicrobial activity and protected cells from oxidative stress damage in diabetic wounds [44].

From the EDX mapping in Figure 2, the elements were uniformly distributed, except for Cr, which did not completely dissolve into a solid solution. Micro-segregation of metallic elements has been ascribed to several reasons, including the difference in melting point, atomic size difference, and mixing enthalpy of binary atoms [24,38]. Plausibly, the mixing enthalpy ($∆H_{AB}$) of binary atoms (*A, B*) may be influential in the case of HEA CrMoNbTiZr: Nb–Ti ($∆H_{NbTi}=$ 2 kJ/mol), Nb–Zr ($∆H_{NbZr}=$ 3.9 kJ/mol), and Cr–Mo ($∆H_{CrMo}=$ 0.4 kJ/mol). Therefore, Cr and Nb were segregated from the BCC solid solution to form other enriched phases. Additionally, since no prior mechanical milling was performed for alloying in this work, the SPS sintering time may not have been sufficient to facilitate the complete diffusion of the different atoms into a solid solution. Homogeneous heat treatment may be used to eliminate this segregation. Nevertheless, the overall compositional mapping of the elements was very close to the starting composition of equiatomic CrMoNbTiZr HEA, as shown in Table 1.

Some previous studies proposed some empirical parameters to predict the formation of solid solutions in HEAs. These parameters include atomic size difference (*δ*), enthalpy of mixing (Δ*H_mix_*), valence electron concentration (*VEC*), and entropy of mixing (Δ*S_mix_*) [45,46]. Accordingly, Zhang et al. [47] proposed the following quantitative criteria for the formation of a simple solid solution: 12.0 J/(K mol) $<∆S_{mix}$ < 17.5 J/(K mol); −15.0 kJ/mol $<∆H_{mix}<$ 5.0 kJ/mol; and δ < 6.5%. Similarly, Guo and Liu proposed the limits 0 ≤ *δ* ≤ 8.5%; −22.0 kJ/mol ≤ $∆H_{mix}$≤ 7.0 kJ/mol; and 11.0 ≤ $∆S_{mix}$ ≤ 19.5 J/(K mol) [48]. A *VEC* ≥ 8.0 predicts the solid solution to be FCC; mixed FCC and BCC phases will appear when 6.87 ≤ VEC < 8.0, and a sole BCC phase is expected when VEC < 6.87 [49]. While some discrepancies exist in these limits, they are still helpful in providing insights into solid solution formation in HEAs. Calculations using the above parameters showed that the equiatomic CrMoNbTiZr HEA had $∆H_{mix}$ = −5.8 kJ/mol; $∆S_{mix}$ = 13.4 J/(K mol); and δ = 8.2; which satisfied the criteria for the formation of solid solutions. Additionally, the calculated *VEC* of the alloy was 5.0, which was less than 6.87, thus showing the propensity of the HEA to form a stable BCC structure, which agreed with both CALPHAD and experimental results.

### 3.2. Microhardness and Compression Characteristics

The global Vickers microhardness of the HEA was 531.5 ± 99.7 HV. The hardness HV exceeds the reported hardness for Ti0.5ZrNbTaMo (500 HV) [50], TiZrHfNbTa (320 HV) [51], and Ti6Al4V (320 HV) [50]. CrMoNbTiZr HEA showed more than one phase in the microstructure; therefore, within the limits of the resolution of the optical microscopy, the microhardness of the two identifiable “light” and “dark” phases was examined and found to be 364.7 ± 99.4 and 641.8 ± 63.0 HV, respectively. Due to the difference in the microhardness of the two phases, the harder phase (dark) was assigned to the Laves phase, while the other was assigned to the BCC phase. Previous studies have shown that the Laves phase has a higher hardness than the BCC phase [38,39,40], while the BCC phase has higher hardness than the FCC phase [52]. A study on the AlCoCrFeNbxNi HEA system demonstrated that hardness increased with increasing Nb content as the crystal structure transformed from the BCC (520 HV) to the BCC + Laves phase (655 HV) [38]. A similar enhancement in the microhardness was reported for the CoCrCuFeNiNb HEA coating, where Nb addition induced the crystallization of the Laves phase [39].

**Figure 3:**
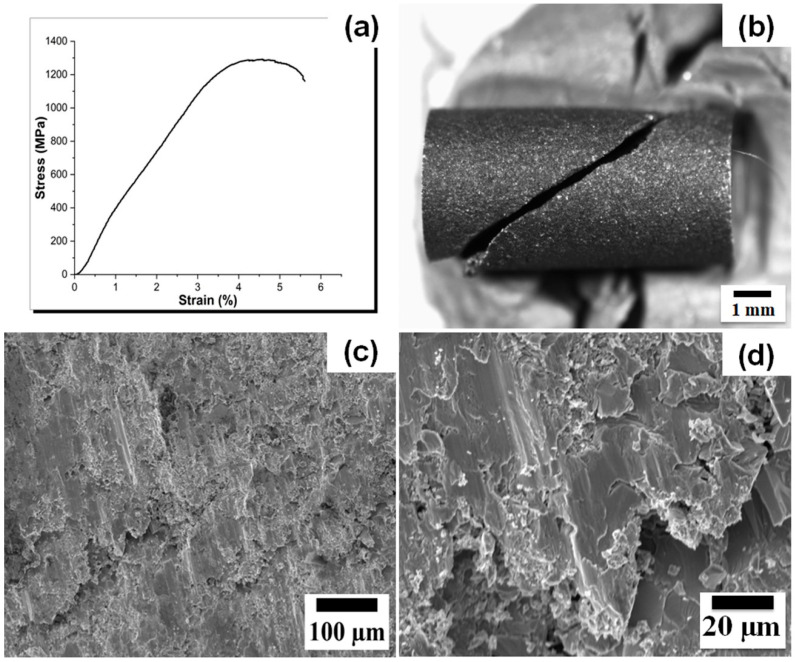
(**a**) Representative room temperature compression stress–strain curve. (**b**) Typical macro-image of a test sample showing 45° fracture relative to the compression axis. (**c**,**d**) SEM morphology of fracture surface with cleavage bands.

The room-temperature compression stress–strain curve is presented in Figure 3a. The CrMoNbTiZr HEA exhibited a linear elastic behavior in the early compression stage, followed by yielding, which progressed into the ultimate compressive strength. After that, strength decreased with increasing strain, and the sample fractured at about 5.6% plastic strain ($\varepsilon_{p}$). The average ultimate compressive strength ($\sigma_{UCS}$) and compressive yield strength ($\sigma_{YS}$) were 1235.7 ± 42.8 MPa and 1110.8 ± 78.6 MPa, respectively. The average compression modulus ($E_{c}$) was estimated to be 34.9 ± 3.0 GPa. Compared to similar refractory-based HEA, these compressive characteristics are higher than those of equiatomic NbMoTaW ($\sigma_{UCS}$ = 1211 MPa; $\sigma_{YS}$ = 1058 MPa; $\varepsilon_{p}$ = 2.1%) [53] and equiatomic TiZrHfNbTa ($\sigma_{UCS}$ = 878 MPa; $\sigma_{YS}$ = 834 MPa) [51]. The $E_{c}$ of the HEA alloy in this study is lower than the $E_{c}$ of Ti alloys (59–142 GPa) [54,55], 316L stainless steel (210 GPa) [8] and Cr–Co alloys (240 GPa) [8,56], and similar to the range measured for human cortical bone (10–30 GPa) [36]. Therefore, the problem of stress-shielding or loosening of Ti-based implants [8,9] and consequent revision surgery [8] may be minimized with the use of CrMoNbTiZr HEA.

CrMoNbTiZr HEA fractured at approximately 45° to the compression axis, as shown in the macroscopic image in Figure 3b. The fracture surface is rough, with several cleavage bands. A higher magnification image (Figure 3d) shows that the cleavage bands are characterized by flat facets, faceted steps, and river-like patterns. These features are consistent with quasi-cleavage fracture [53], which is related to the crystal structure. CrMoNbTiZr HEA has a mixture of BCC, Laves, and carbides, which are characterized by high hardness. In the BCC crystal structure, there is a limited number of slip systems, and the critical stress required to actuate the slip system is higher than in an FCC structure [57]. Similarly, the presence of carbides, in the form of C in the BCC interstices, has also been reported to reduce plasticity [27]. Therefore, the alloy exhibited high strength with minimal plasticity. In a Ti6Al4V alloy subjected to compression testing at room temperature, similar shear bands and fracture at 45° were observed and ascribed primarily to the state of stress. It was adduced that once the maximum stress was attained, tensile stress formed in the middle of the specimen was responsible for the void formation and crack propagation along the shear bands. Plastic deformation occurred on the planes of maximum shear stress, which were inclined at 45° to the compression axis [58].

### 3.3. Potentiodynamic Analyses

**Figure 4:**
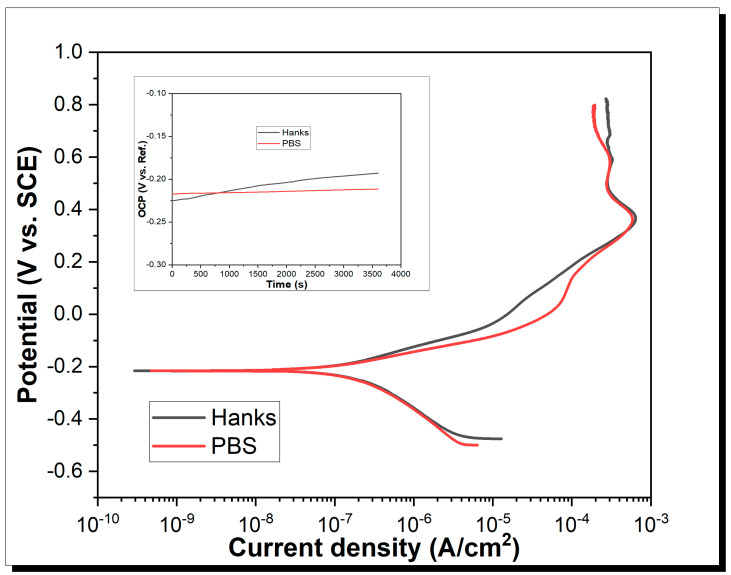
Potentiodynamic curve of CrMoNbTiZr sample in Hanks and PBS.

Potentiodynamic polarization curves of CrMoNbTiZr HEA in PBS and Hanks physiological solutions at room temperature are shown in Figure 4. The corrosion current density ($i_{corr}$) and corrosion potentials ($E_{corr}$), which were estimated from Tafel extrapolation, are shown in Table 2. From Figure 4, as corrosion potential increases, the anodic part of the polarization curve shows two regions: an anodic dissolution and a passivation region. $i_{crit}$ (or $E_{crit}$) is the critical corrosion density (or equivalent critical potential) where the alloy begins to passivate due to the formation of a protective surface film [50,51]. At this point, an increase in the applied potential did not increase the measured current density. $i_{crit}$ in PBS (600 µA/cm^2^) was slightly lower than in Hanks’ (650 µA/cm^2^), indicating that passivation occurred faster in PBS. Complete surface passivation occurred at 907.25 and 906.83 mA/cm^2^, respectively, in PBS and Hanks solutions. At this point, a further increase in potential did not result in any measurable current density. Overall, the corrosion rate (Cr) in PBS (1.24 × 10^−3^ mm/yr) was lower than in Hanks’ solution (2.03 × 10^−3^ mm/yr).

The primary passive potential ($E_{pass}$) is quite far from the $E_{corr}$, suggesting that passivation was not instantaneous, although an almost immediate passivity was reported for TiZrHfNbTa HEAs [51] in Hanks and Al0.1CoCrFeNi in 3.5 wt.% NaCl [59]. Nevertheless, the corrosion current density of CrMoNbTiZr (0.14 µA/cm^2^) is lower than the values reported for some commonly used biomedical alloys, such as CoCrMo in PBS (18.57 µA/cm^2^) [60], wrought 316L in PBS (0.60 µA/cm^2^), and additively manufactured 316L in PBS (0.57 µA/cm^2^) [61]. A summary of the potentiodynamic characteristics of the HEA is shown in Table 2.

**Figure 5:**
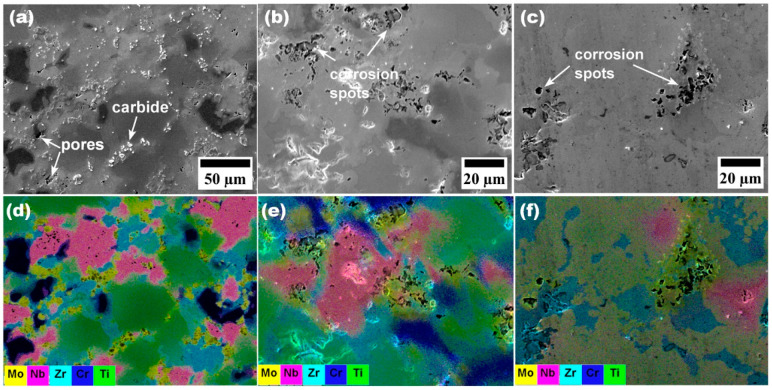
(**a**) SEM image of CrMoNbTiZr before potentiodynamic polarization test. (**b**) Microstructure after corrosion in Hanks. (**c**) Microstructure after corrosion in PBS. Corresponding EDX analyses of the microstructural images of (**a**–**c**) are shown in (**d**–**f**), respectively.

SEM images (Figure 5b,c) of the corroded surface showed numerous localized corrosion spots. Although the potentiodynamic polarization curve did not indicate the occurrence of the pitting phenomenon, the pores on the as-sintered surface of the sample (Figure 5a) seemed to play the role of crevices, which resulted in localized corrosion at the pores. Pores and other surface asperities generally possess high free energies, which can exacerbate corrosion. Similar pore-induced corrosion pits have been observed on SPS 316L samples in NaCl solution [62]. The pores may be eliminated and further densify the alloy by a subsequent processing step using hot isostatic processing. EDX analysis of the corroded surfaces in both PBS and Hanks solutions showed that localized corrosion occurred within the Mo-rich regions. According to the electrochemical series, the standard electrode potential of Mo is the lowest of the other elements comprising the HEA (Cr^3+^ + 3 e^−^ ⇌ Cr(s), $E^{0}$ = −0.74 V); Mo^3+^ + 3 e^−^ ⇌ Mo(s), $E^{0}$ = −0.20 V; Nb^3+^ + 3 e^−^ ⇌ Nb(s), $E^{0}$ = −1.099; Ti^3+^ + 3 e^−^ ⇌ Ti(s), $E^{0}$ = −1.37 V; Zr^4+^ + 4 e^−^ ⇌ Zr(s), $E^{0}$ = −1.45 V) [63].

### 3.4. Wear Resistance of CrMoNbTiZr HEA

**Figure 6:**
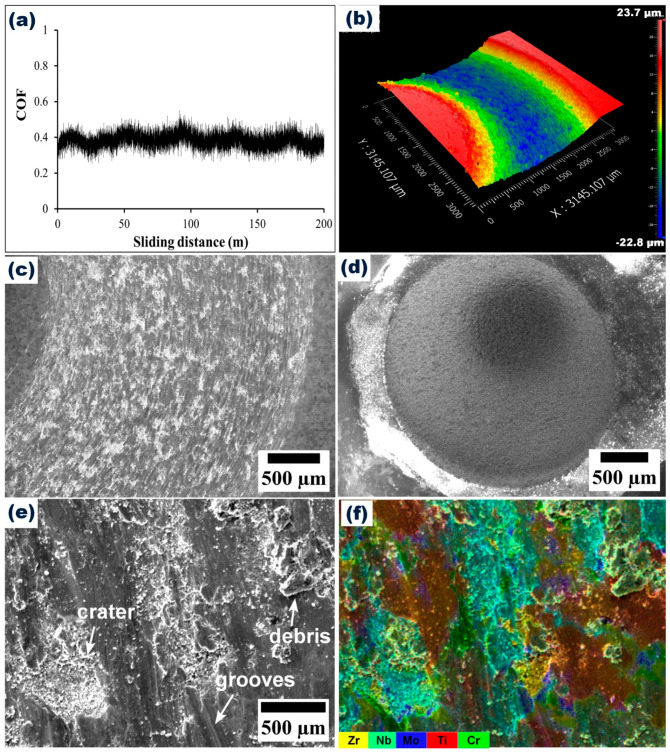
(**a**) COF plot. (**b**) Profilometry of wear track. (**c**) SEM morphology of wear track. (**d**) SEM morphology of Al_2_O_3_ counter ball. (**e**) Higher magnification of wear track in (**c**). (**f**) EDX mapping of wear track in (**e**).

Figure 6a shows the variation of the coefficient of friction (COF) of the CrMoNbTiZr HEA against the alumina ball. The average COF was 0.43 ± 0.06. Wear track morphology and profilometry are shown in Figure 6b. The wear morphology was characterized by grooves and a compacted layer of wear debris, indicative of abrasive and adhesive wear [64] and repeated cycles of wear track deformation [65], respectively. The formation of wear groves has been attributed to repetitive ploughing of the wear track from the wear debris [64]. As shown in Figure 6d, the wear scar on the alumina ball was 2.8 mm in diameter due to the induced wear and deformation from the HEA substrate (Figure 6c). Alumina has a Vickers microhardness of about 1365 HV, which exceeds the value recorded for the CrMoNbTiZr HEA. Hence, it exhibited minimal pick-up of elements from the HEA substrate, as indicated in the EDX analysis of the ball surface (Point 1 in Table 3). The oxygen content on the worn surface of CrMoNbTiZr HEA (Point 3) was about twice that on the alloy surface (Point 2), suggesting that oxidation occurred during the wear process, with Ti and Zr being the likely oxidized components due to their higher concentration in the wear scar (Point 3). Optical profilometry of the wear track (Figure 6b) shows that the scar is about 47 µm deep, while the average wear rate was estimated to be 3.4 × 10^−4^ mm^3^/Nm, which was lower than the 4.0 × 10^−3^ mm^3^/Nm for CuMoTaWV [20] and 3.5 × 10^−4^ mm^3^/Nm for a typical biomedical Ti6Al4V alloy [50].

This result indicates the high wear resistance of the HEA. The enhanced wear resistance in this study may be associated with the high microhardness of the BCC and Laves phases. Moreover, in situ reinforcing carbides of Nb (NbC) and Ti-Mo (Ti_0.5_Mo_0.5_C) are also expected to enhance the wear resistance of the alloy, as observed in some earlier reports [40,64]. Highly wear-resistant materials are required for orthopedic applications to minimize debris accumulation [6] and metal allergy [7] due to the accumulation of metallic ions from wear debris.

The totality of the experimental results and discussions indicate that microstructural design through the selection of the constituent elements greatly influenced the microhardness, compressive and wear properties of the alloy. It can be concluded that the mechanical, bio-corrosion, and wear-resistance properties of the CrMoNbTiZr HEA developed in this study showed a good propensity for application as a biomaterial.

## 4. Conclusions

A novel equiatomic CrMoNbTiZr HEA was synthesized by elemental powder mixing and consolidated by spark plasma sintering. The relative density of the alloy approached 97% of the theoretical density, indicating the suitability of the spark plasma sintering technique to produce relatively dense material. The microstructure, mechanical properties, and bio-corrosion behavior of the HEA were investigated. The microstructure consisted primarily of BCC and Laves phases, which matched the prediction from a thermodynamic CALPHAD simulation. During sintering, carbides of Nb and Mo were formed due to the diffusion of carbon from the graphite die. The HEA exhibited a global Vickers microhardness of 531.5 ± 99.7 HV, while the individual BCC and Laves phases had values of 364.7 ± 99.4 and 641.8 ± 63.0 HV, respectively. The ultimate compressive strength and compressive yield strength were 1235.7 ± 42.8 MPa and 1110.8 ± 78.6 MPa, respectively. Its elasticity modulus of 34.9 ± 3.1 GPa was significantly lower than the values reported for commonly used biomaterials made from Ti-based, Cr–Co-based, and stainless-steel alloys. Importantly, it was well within the range reported for cortical bone. The alloy exhibited good bio-corrosion resistance with low current densities of 0.14 and 0.24 µA/cm^2^ in PBS and Hanks’ solutions. These translated to low corrosion rates of 1.24 × 10^−3^ and 2.03 × 10^−3^ mm/yr in PBS and Hanks’ solutions, respectively. CrMoNbTiZr HEA exhibited an average COF of 0.43 ± 0.06, characterized mainly by abrasive and adhesive wear mechanisms. The totality of these results showed that microstructural design through the selection of the constituent elements greatly influenced the mechanical and electrochemical properties of the alloy. The HEA developed in this study showed a good propensity for application as a biomaterial due to good mechanical properties and excellent bio-corrosion resistance. Finally, further research is required to investigate the fretting wear behavior and assess other in vitro and in vivo cell proliferation properties of the alloy.

## Figures and Tables

**Table 1:** EDX point analysis of the different phases in CrMoNbTiZr HEA in at.%.

Regions	Cr	Mo	Nb	Ti	Zr
BCC (dark gray)	7.42 ± 1.96	12.08 ± 1.71	8.29 ± 4.77	51.97 ± 3.661	20.40 ± 5.53
Laves (light gray)	2.84 ± 1.43	-	79.87 ± 24.26	2.94 ± 2.26	-
Carbide	2.35 ± 1.06	-	96.68 ± 5.19	4.50 ± 0.00	-
X	98.10 ± 0.44	1.80 ± 0.00	-	0.98 ± 0.61	-
Overall composition	20.85 ± 1.06	19.60 ± 0.14	17.65 ± 1.48	20.50 ± 0.99	21.40 ± 1.41

**Table 2:** Parameters extracted from potentiodynamic polarization curve.

	OCP (mV vs. SCE)	$E_{corr}$ (mV vs. SCE)	$i_{corr}$ (µA/cm^2^)	Cr (mm/y)	$i_{pass}$ (mA/cm^2^)	$i_{crit}$ (µA/cm^2^)	$E_{crit}$ (V/SCE)
PBS	−200.17	−216	0.14	1.24 × 10^−3^	907.25	600	0.36
Hanks	−176.25	−215	0.24	2.03 × 10^−3^	906.83	650	0.36

**Table 3:** EDX point analysis of elemental concentration (at.%) of points 1–3 in Figure 6.

Substrate	EDX Point	Cr	Mo	Nb	Ti	Zr	Al	O
Ball	1	0.5	1.4	1.7	0.8	1.9	17.6	75.9
Bare	2	19.6	14.9	15	17.6	16.6	-	16.3
Track	3	8.6	10.4	8.9	22.9	14.3	3.1	31.9

## Data Availability

Data will be made available upon reasonable request.
